# Osteoinductive Electrospun Scaffold Based on PCL-Col as a Regenerative Therapy for Peri-Implantitis

**DOI:** 10.3390/pharmaceutics15071939

**Published:** 2023-07-12

**Authors:** Claudia Sanhueza, Jeyson Hermosilla, Catherine Klein, Alejandra Chaparro, Iván Valdivia-Gandur, Víctor Beltrán, Francisca Acevedo

**Affiliations:** 1 Center of Excellence in Translational Medicine-Scientific and Technology Bioresource Nucleus (CEMT-BIOREN), Faculty of Medicine, Universidad de La Frontera, Temuco 4780000, Chile; 2 Doctorado en Ciencias de Recursos Naturales, Universidad de La Frontera, Casilla 54-F, Temuco 4780000, Chile; 3 Department of Periodontology, Center for Biomedical Research, Faculty of Dentistry, Universidad de Los Andes, Av. Plaza 2501, Las Condes, Santiago 7620157, Chile; 4 Biomedical Department, Universidad de Antofagasta, Avenida Angamos 601, Antofagasta 1270300, Chile; 5 Clinical Investigation and Dental Innovation Center (CIDIC), Dental School, Universidad de La Frontera, Temuco 4780000, Chile; 6 Department of Basic Sciences, Faculty of Medicine, Universidad de La Frontera, Casilla 54-D, Temuco 4780000, Chile

**Keywords:** cholecalciferol, polycaprolactone, electrospinning, bone regeneration

## Abstract

Peri-implantitis is a serious condition affecting dental implants that can lead to implant failure and loss of osteointegration if is not diagnosed and treated promptly. Therefore, the development of new materials and approaches to treat this condition is of great interest. In this study, we aimed to develop an electrospun scaffold composed of polycaprolactone (PCL) microfibers loaded with cholecalciferol (Col), which has been shown to promote bone tissue regeneration. The physical and chemical properties of the scaffold were characterized, and its ability to support the attachment and proliferation of MG-63 osteoblast-like cells was evaluated. Our results showed that the electrospun PCL-Col scaffold had a highly porous structure and good mechanical properties. The resulting scaffolds had an average fiber diameter of 2–9 μm and high elongation at break (near six-fold under dry conditions) and elasticity (Young modulus between 0.9 and 9 MPa under dry conditions). Furthermore, the Col-loaded scaffold was found to decrease cell proliferation when the Col content in the scaffolds increased. However, cytotoxicity analysis proved that the PCL scaffold on its own releases more lactate dehydrogenase into the medium than the scaffold containing Col at lower concentrations (PCL-Col A, PCL-Col B, and PCL-Col C). Additionally, the Col-loaded scaffold was shown to effectively promote the expression of alkaline phosphatase and additionally increase the calcium fixation in MG-63 cells. Our findings suggest that the electrospun membrane loaded with Col can potentially treat peri-implantitis by promoting bone formation. However, further studies are needed to assess the efficacy and safety of this membrane in vivo.

## 1. Introduction

Peri-implantitis (PI) is a mainly infectious pathology characterized by inflammation of the peri-implant, which supports connective tissues and the progressive loss of the supporting bone of the osseointegrated implants. It is not an uncommon occurrence, and if it is not diagnosed and treated in time, it can cause the loss of the implant [1,2]. More than 12 million dental implants are installed annually and, according to PI prevalence estimates, around 1 million of these cases will develop PI, which involves the loss of the implant and the bone tissue to which it is osseointegrated [3].

In dentistry, bone grafts or substitutes are mainly used with resorbable and non-resorbable membranes as guided bone regeneration (GBR) mechanisms [4]. Most of the biomaterials proposed in this field focus on developing bone graft-type biomaterials with osteoinductive cells [5].

In the field of bone regeneration, the ideal material for the regenerative treatment of PI must have the following characteristics: (1) possess sufficient mechanical strength to withstand the load to which it will be subjected initially and during the regeneration process; (2) possess adequate permeability and porosity to allow bone regeneration around the implant and in turn avoid infiltration of inflammatory cells in the bone defect that is regenerating; (3) avoid the formation of a bacterial biofilm on the implant surface; and (4) be resorbable and synchronized with the generation of new bone around the implant [6].

Electrospinning is a commonly employed method for fabricating scaffolds. It involves an electrodynamic process, where a solution containing polymers is spun by subjecting it to a high-voltage electric field, resulting in the formation of fibers with varying diameters [7]. The size of the fibers can be modified by manipulating different factors, such as solution characteristics like viscosity, conductivity, surface tension, molecular weight, concentration, and polymer structure. Additionally, process variables, such as electric potential, flow rate, distance between the needle and collector, and the shape and composition of the collector, can also have an impact on the diameter of the fibers. Moreover, environmental parameters such as temperature and humidity should also be considered in the fiber production process. By carefully controlling these parameters, a wide range of fiber diameters can be achieved [8]. This technique allows one to customize and fine-tune the structural and mechanical properties according to the specific requirements of various applications [9].

Ideally, when it comes to tissue engineering, the biomaterials used should be able to mimic the structural and biochemical functions of the extracellular matrix (ECM). Consequently, scaffolds utilized in tissue engineering are often constructed using biodegradable polymeric materials [10]. In this context, polycaprolactone (PCL) emerges as a hydrophobic semi-crystalline polyester that possesses remarkable mechanical strength, high elasticity, biodegradability, non-toxicity, and excellent biocompatibility. Furthermore, PCL has the ability to closely resemble the extracellular matrix (ECM), making it an ideal choice for tissue engineering applications [11]. Specifically, PCL is one of the most extensively studied polymers for its potential applications in various biomedical fields. It has shown promise in areas such as cardiovascular grafts, drug delivery systems, dental splints, bone scaffolds, and wound dressing membranes. PCL’s versatility and favorable properties make it a highly sought-after material for biomedical applications [12]. 

Collagen membranes are the current gold standard in treating PI since they prevent the infiltration of the gingival tissue into the bone defect; however, none of these membranes can stimulate the formation of new bone. Recent studies have shown advances in the technological development of scaffolds conjugated with biomolecules, constituting a potential engineering strategy in tissue regeneration [4].

**Figure 1:**
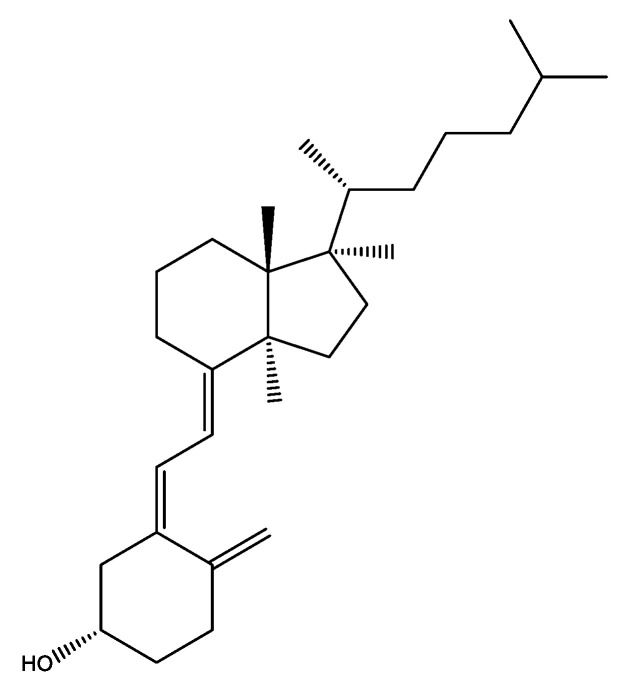
Cholecalciferol (Col) chemical structure.

Cholecalciferol (Col), or D3 as it is commonly called, is a lipophilic molecule with low solubility in water (Figure 1). The primary function of vitamin D is to maintain calcium and phosphorus homeostasis in the body, which is relevant for bone mineralization and thus prevents skeletal diseases like rickets. Col is usually transported to the liver, where it is hydroxylated at C25 and then transported to the kidney, where it is hydroxylated at C1 and converted to 1,25 (OH) 2D3, which is the active form of vitamin D. However, evidence has shown that there may be extra renal synthesis of 1,25 (OH) 2D3, where tissues such as skin, liver, and a variety of hematopoietic cells, bone cells, and tissues are included [13]. However, little is known about the effect of Col on bone regeneration in the dental field and its encapsulation into membranes for tissue engineering.

In this context, this study aimed to develop an electrospun scaffold composed of polycaprolactone microfibers (PCL) loaded with Col for its potential use in the treatment of peri-implantitis. To achieve this, various electrospinning conditions were experimented with, including adjusting the polymer and Col concentrations, the voltage applied, and flow rate. These parameters were systematically varied and tested to optimize the electrospinning process and achieve the desired characteristics for the PCL membranes loaded with Col. After the scaffold fabrication process was completed, a comprehensive chemical characterization was carried out. Subsequently, the proliferation and cytotoxicity of MG-63 cells were evaluated, along with the assessment of calcium fixation within the membranes. This was achieved by loading the PCL membranes with various concentrations of Col.

## 2. Materials and Methods

### 2.1. Materials

Polycaprolactone (PCL) (80 kDa) and Alizarin Red S were purchased from Sigma Aldrich Co. (St. Louis, MO, USA). The alkaline phosphatase assay kit (BCIP-NBT), cholecalciferol (Col), dexamethasone, potassium phosphate monobasic, ascorbic acid, chloroform, and formaldehyde were purchased from Merck (Darmstadt, Germany). Dubelcco’s phosphate saline buffer (DPBS), α-MEM medium, fetal bovine serum (FBS), penicillin and streptomycin, and Trypsin-EDTA were obtained from HyClone (Logan, UT, USA). The Alamar blue assay and CyQUANT LDH cytotoxicity assay kit were purchased from Invitrogen, ThermoFisher (Waltham, MA, USA).

### 2.2. Electrospinning Parameters

The impact of voltage, flow rate, and polymer concentration on the diameter and morphology of the fibers was assessed at three distinct levels. Initially, PCL solutions with concentrations of 8–18% (by weight) were prepared using chloroform as the solvent. For each polymer solution, three different distances between the needle and the collector (10 cm, 15 cm, and 20 cm) and three voltage settings (15 kV, 20 kV, and 25 kV) were examined.

The prepared solutions were loaded into 10 mL plastic syringes, connected to #8 needles with an inner diameter of 0.4 mm. A syringe pump was utilized to control the flow rate and deliver the polymer solutions through the needle tip. The electrospinning process was carried out at room temperature, employing a rotating drum collector set at 100 rpm. The distance between the needle tip and the collector was fixed at 15 cm. All the generated fibers were collected on aluminum foil that covered the grounded drum collector. 

Once the electrospinning working conditions were established, Col was incorporated into the PCL/chloroform working solution. Different concentrations of Col loaded in the fibers were evaluated. The fiber diameter and morphology index were determined by analyzing micrographs obtained using a scanning electron microscope (SEM, Hitachi SU2500 model). The images captured were analyzed with the ImageJ software version 1.51k, allowing for precise measurements of their diameter, as well as the evaluation of their overall morphology.

### 2.3. Physicochemical Characterization

Physicochemical characterization was carried out in accordance with the protocols conducted in previous studies developed by our research group [14,15,16].

#### 2.3.1. Mechanical Properties

The tensile modulus (E), elongation at break (ε), and tensile strength (σ) of the electrospun scaffolds were determined by evaluating scaffolds that were cut into pieces measuring 4.0 × 1.0 cm^2^. The measurements were performed using a CT3-10KG00000084 digital texture analyzer from Brookfield, UK. The analysis was conducted with a crosshead speed of 0.1 mm/s and a gap of 8.0 mm, allowing for the assessment of mechanical properties such as the stiffness (tensile modulus), stretchability (elongation at break), and strength (tensile strength) of the electrospun scaffolds. All the tensile tests were conducted at room temperature.

#### 2.3.2. TGA-DSC

Thermogravimetric analysis (TGA) and differential scanning calorimetry (DSC) were performed on the scaffolds weighing approximately 20 mg. The analysis was conducted using a DSC model STA 6000 from Perkin Elmer Inc. (Waltham, MA, USA). The measurements were carried out under a nitrogen atmosphere with a flow rate of 40 mL/min. The samples were heated from 25 to 600 °C at a constant heating rate of 15 °C/min. The TGA allowed for the determination of the thermal stability and decomposition behavior of the scaffolds, while the DSC provided insights into their thermal properties, e.g., melting point.

#### 2.3.3. FTIR

The chemical structure of the scaffolds loaded with Col was analyzed using Fourier-transform infrared (FTIR) spectrometry. The FTIR measurements were performed using a Cary 630 FTIR Spectrometer from Agilent Technologies Inc. (Danbury, CT, USA). The software Resolution Pro version 2.20.06 was employed for data analysis. A scanning range of 4000–600 cm^−1^ was used to obtain the FTIR spectra. 

#### 2.3.4. Water Contact Angle

The water contact angle measurement was conducted using custom-made equipment specifically designed for this purpose. The equipment consisted of a setup where the samples, cut into 2 cm × 2 cm dimensions, were placed on a level test surface. A camera was employed to capture high-resolution images of the samples. A droplet of purified water was carefully placed on the surface of each sample, and the resulting contact angle was measured using ImageJ software version 1.51k to analyze the images and determine the angle accurately.

### 2.4. Biocompatibility Assays

#### 2.4.1. Proliferation Analysis

MG-63 osteoblast-like cell lines were cultured in DMEM and α-MEM expansion medium, respectively. Both media used in the experiment were supplemented with 10% (*v*/*v*) fetal bovine serum (HyClone, USA) and 1% (*v*/*v*) penicillin and streptomycin (HyClone, USA). The cells were maintained at a temperature of 37 °C in a humidified atmosphere with 95% humidity and 5% CO_2_. When the flasks reached approximately 80–90% confluency, indicating that the cells had grown and covered most of the culture surface, they were sub-cultured. To detach the cells from the tissue culture surface, they were exposed to 0.25% (*w*/*v*) trypsin-EDTA (Corning, Singapore) solution for three minutes.

Cell proliferation in the presence of PCL scaffolds loaded with various concentrations of Col was evaluated using the Alamar blue assay, following the manufactures recommendations. Briefly, the scaffolds were prepared by cutting them into 0.6 cm diameter pieces and then sterilized with UV radiation on both sides for 30 min. Subsequently, the sterilized scaffolds were placed into 96-well microplates. Each well was seeded with 20,000 cells in 200 µL of culture media and incubated at 37 °C. The experiments were conducted in triplicate for each treatment and control group. Cell viability was assessed after 1, 3, and 7 days of incubation. The media were removed, and the cells were washed with 200 µL of DPBS/Modified solution for 5 min to remove phenol red from the culture media. Following the wash, 150 µL of Alamar blue^TM^ solution (diluted 1:10 in phenol red-free DMEM) was added to each well and incubated for 1.5 h. Subsequently, 100 µL of the solution was transferred to a black 96-well plate, and fluorescence was measured using a BIOTEK, Synergy H1 Hybrid reader (Agilent technologies, College Park, MD, USA) at an excitation wavelength of λ_ex_ 546 nm and an emission wavelength of λ_em_ 590 nm.

#### 2.4.2. Cytotoxicity Analysis

The lactate dehydrogenase CyQUANT LDH cytotoxicity assay kit (Invitrogen, Carlsbad, CA, USA) was used according to the manufacturer’s instructions. Briefly, the lactate dehydrogenase (LDH) released into the medium was quantified. The culture medium was set aside to measure extracellular LDH activity. To estimate the intracellular LDH activity, the cells were lysed using the assay buffer provided in the kit. The assay buffer facilitated the release of intracellular LDH, allowing for its activity to be measured. The SPECTROStar Nano Microplate Reader (BMG LABTECH, Ortenberg, Germany) was used to detect the OD values at 490 and 680 nm. 

% Cytotoxicity was calculated using the following formula:

(1) $$\%\;\mathrm{Cytotoxicity}=\frac{\left(\mathrm{Compound}\;\mathrm{treated}\;\mathrm{LDH}\;\mathrm{activity}-\mathrm{DMEM}\;\mathrm{media}\;\mathrm{LDH}\;\mathrm{activity}\right)}{\left(\mathrm{Lysed}\;\mathrm{cells}\;\mathrm{LDH}\;\mathrm{activity}-\mathrm{DMEM}\;\mathrm{media}\;\mathrm{LDH}\;\mathrm{activity}\right)}$$

At least 3 replicates were conducted in each experiment.

#### 2.4.3. Hemocompatibility Evaluation

The use of fresh human whole blood from anonymous healthy donors was approved by the ethics committee at the Universidad de La Frontera (File N°099_20). The method was conducted according to the process reported by Sanhueza et al. [17]. The blood was diluted with a 0.9% (*w*/*v*) NaCl solution at a ratio of 1:30. Scaffold pieces weighing approximately 5 mg each were placed individually in Eppendorf tubes containing 1 mL of the diluted blood solution. Simultaneously, negative control samples were prepared using 100 μL of saline solution, while positive control samples were prepared using 100 μL of Triton X-100 at a concentration of 4% (*v*/*v*). The experiments were conducted in triplicate. The Eppendorf tubes were placed on an orbital shaker set at 100 rpm and maintained at a temperature of 37 °C. After 60 min of incubation, 1 mL of the suspension was removed from each tube and centrifuged at 10,000× *g* for 10 min. The absorbance of the supernatant (100 μL) was measured at 540 nm using a plate reader. The percentages of hemolysis were calculated using the following formula:

(2) $$\%\;\mathrm{hemolysis}=\frac{\mathrm{Abs}-{\;\mathrm{Abs}}_{0}}{{\mathrm{Abs}}_{100}-{\;\mathrm{Abs}}_{0}}$$

In the above-listed equation, Abs_100_ represents the absorbance of the positive control, and Abs_0_ represents the absorbance of the negative control. These values are used in the calculation to determine the percentages of hemolysis.

#### 2.4.4. Bone Matrix Evaluation

To assess the in vitro osteogenic activity of the MG-63 cell line, alkaline phosphatase (ALP) activity, extracellular matrix deposition, and the mineralization of the extracellular matrix were measured. The cells were seeded on the samples and cultured in α-MEM expansion medium for 14 days, during which they were exposed to osteogenic medium. The osteogenic medium (OM) is designed to promote osteogenic differentiation and enhance the expression of osteogenic markers in the cultured cells. The ALP activity serves as an indicator of early osteogenic differentiation, while measuring extracellular matrix deposition and mineralization provides insights into the progression of osteogenesis and the formation of mineralized bone-like tissue by the MG-63 cells. The medium was removed every 2–3 days and changed for fresh medium. The OM consisted of dexamethasone 100 nM, potassium phosphate monobasic 30 mM, and ascorbic acid 50 µg/mL. 

The alkaline phosphatase activity was measured using an alkaline phosphatase assay kit (BCIP-NBT) in accordance with the process reported by Pereira et al. [18] (with some modifications). The assay used in this study is based on a chromogenic reaction that is initiated by the cleavage of the phosphate group of BCIP (5-bromo-4-chloro-3′-indolyphosphate p-toluidine) dihydrogen phosphate by the alkaline phosphatase enzyme present in the cells. This enzymatic reaction generates a proton, which then reduces nitro-blue tetrazolium chloride (NBT). As a result, an intense and insoluble black-purple precipitate is formed. The formation of this precipitate serves as a visual indicator of alkaline phosphatase activity in the cells, providing a quantitative measure of the osteogenic activity of the MG-63 cell line. After 7 and 14 days of incubation, wells were carefully washed twice with PBS. Then, 500 µL of the BCIP-NBT solution was added to each well, and the plate was incubated for 2 h at 37 °C. The black-purple precipitate was then solubilized in 500 µL of isopropanol per well. Subsequently, 100 µL of the solution from each well was transferred in duplicate to a flat-bottomed 96-well plate. The optical density of the solution was measured at 595 nm using a plate microreader.

The Alizarin Red S assay (ARS, Sigma-Aldrich) was employed to assess the calcium deposition achieved by the MG-63 cells on culture day 7 on OM. The methodology described by Gregory et al. [19] was followed with some modifications. First, the samples were fixed using a 37% formaldehyde solution in PBS pH 7.4. Subsequently, they were immersed in a 40 mM solution of Alizarin Red S for 30 min. After the staining step, the samples underwent a series of washes using PBS buffer. Each wash cycle consisted of a 5 min period of orbital shaking at a speed of 50 rpm. 

In both assays, control samples were included to account for any potential interactions between the samples and the reagents used, as well as the culture medium itself. These control samples were subjected to the same incubation conditions as the experimental samples but did not contain any cells. In the alkaline phosphatase assay, the reported absorbance values correspond to the absorbance of the sample minus the absorbance of its respective cell-free control sample. This control subtraction allows for the specific measurement of the alkaline phosphatase activity that is responsible for the presence of cells in the sample.

### 2.5. Statistical Analysis

All experimental data were obtained from triplicate samples and presented as mean ± standard deviation (SD). Statistical analysis was performed using analysis of variance (ANOVA) to assess significant differences. When a *p*-value was found to be less than 0.05, indicating statistical significance, Tukey’s test was conducted. The statistical analyses were carried out using GraphPad Prism 9 software (San Diego, CA, USA).

## 3. Results and Discussion

### 3.1. Fibers Characterization

The objective of this study was to generate micrometric-sized fibers to fabricate scaffolds with a pore size that facilitates cell infiltration and promotes bone regeneration. To produce micrometric-sized fibers, various electrospinning conditions were investigated. The impact of polymer concentrations, voltage, and the distance between the needle and the collector was examined while maintaining a constant flow rate. Different concentrations of polycaprolactone (PCL) dissolved in chloroform were tested. These specific findings are presented as Supplementary Data (Table S1). Some authors have reported using chloroform in electrospun PCL solutions at different concentrations, and Van der Schueren et al. [20] reported uniform PCL fibers in the order of micro-size solving PCL in chloroform. Based on the screening of electrospinning parameters, the selected conditions for this study were as follows: a PCL concentration of 14 %wt, a flow rate of 2 mL/h, and a voltage of 25 kV.

**Figure 2:**
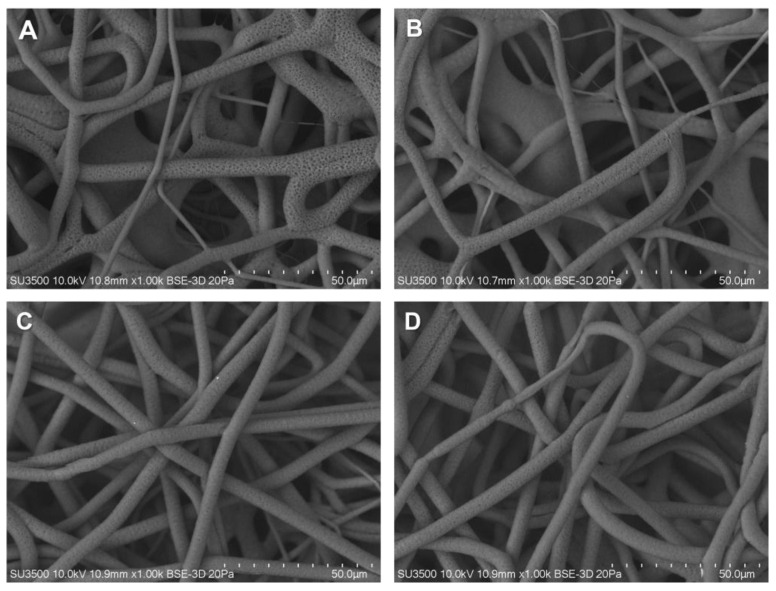
Images obtained from SEM of PCL scaffold containing Col at different concentrations (**A**) PCL-Col A, (**B**) PCL-Col B, (**C**) PCL-Col C y (**D**), PCL-Col D. Each image was conducted at 10 kV and a magnification of 1.00 k.

**Figure 3:**
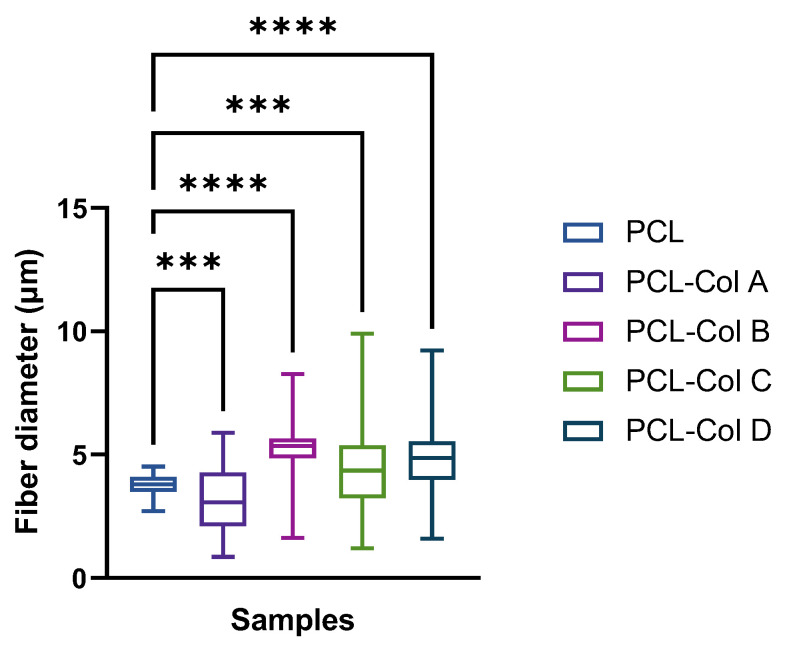
Box and whisker plots representing fiber diameter for PCL scaffolds loaded with different Col concentrations (*** represents a *p* value < 0.001, and **** represents a *p* < 0.0001).

PCL membranes show a distribution of fibers from 2.70 to 4.52 µm, with a median of 3.79 µm. Upon the addition of Col into the membranes, significant differences were observed in the median, and an increase in the fiber diameter distribution was obtained (Figure 2 and Figure 3). Thus, when Col is incorporated into the membranes, the mean fiber diameters were as follows: PCL-Col A was 4.70 ± 1.36 µm; PCL-Col B was 4.48 ± 1.72 µm; PCL-Col C was 5.19 ± 0.94 µm; and PCL-Col D was 3.21 ± 1.21 µm. These measurements indicate that the presence of Col led to an increase in the average fiber diameter compared to the PCL membranes without Col. This change in fiber diameter distribution suggests that the presence of Col affects the electrospinning process, potentially altering the morphology of the resulting membranes.

### 3.2. Mechanical Characterization

In tissue regeneration, it is crucial to have scaffolds with mechanical properties that match the specific requirements of the target tissue. For bone regeneration, the membranes must possess adequate strength to withstand the forces exerted during surgical procedures, as well as those generated by physiological activities and tissue growth. This is essential to ensure the stability and longevity of the scaffold within the body, allowing for successful bone regeneration. Additionally, a strong and stable scaffold can enhance the integration between the implanted scaffold and the surrounding native tissue, promoting proper bone regeneration and functional recovery [21].

Therefore, the PCL scaffolds loaded with different concentrations of Col were evaluated mechanically by using a tension test. In addition, the maximum elongation at break (%), the stress at break (MPa), and the Young’s modulus (MPa) were determined in dry scaffolds and in scaffolds that were soaked overnight in PBS buffer at pH 7.4 at room temperature, since scaffolds may transition from a dry (manufacture and storage) to a wet environment (application) [22]. 

Table 1 presents the obtained values of the PCL scaffolds, and the results of the mechanical properties analysis align with the observed changes in scaffold morphology, as visualized via electron microscopy. In terms of elongation at break, scaffolds for bone tissue regeneration typically exhibit a limited ability to undergo deformation before fracturing, with values close to 1% to 30% [23,24]. However, our study revealed that our scaffolds demonstrated an elongation at break ranging from 500% to 1000%, which is significantly higher than the typical range. This indicates that our scaffolds possess excellent flexibility and deformability, allowing for greater elongation before reaching their breaking point.

Interestingly, as the content of Col increased in the scaffolds, a general decrease in elongation at break was observed, except for the PCL-Col C scaffold. The other scaffolds exhibited a reduction in elongation at break, with values ranging from 665.26% to 589.38% for the PCL and PCL-Col D scaffolds, respectively. This suggests that the incorporation of Col into the scaffolds may slightly compromise their flexibility and limit their ability to undergo deformation.

Regarding tensile strength, scaffolds for bone tissue regeneration typically display values ranging from 1 MPa to 10 MPa [25]. However, for scaffolds intended for load-bearing applications, higher tensile strengths of up to 10 MPa or even exceeding 50 MPa may be observed. In our study, the tensile strength of our scaffolds was determined to be between 1.11 and 3.1 MPa under dry conditions and 12.61 and 25.21 MPa under wet conditions, indicating that they possess sufficient strength to withstand the mechanical demands required for bone regeneration.

Furthermore, the Young’s modulus of scaffolds for bone tissue regeneration varies depending on the materials employed. Generally, the elastic modulus of scaffolds for tissue regeneration falls within a wide range of 1 MPa to 10 GPa. The specific value within this range depends on various factors, including the composition of the scaffold, the blending of different polymers, and the incorporation of bioactive compounds [26,27]. In our study, we measured the elastic modulus of our scaffolds and found that it was 0.97–8.92 MPa in dry conditions and 12.24–35.81 MPa in wet conditions, which aligns with the expected range for the materials used. Similar results were obtained by Castro et al. [28], who developed a PCL scaffold loaded with silica nanoparticles for bone tissue regeneration.

The tensile strength and the Young’s modulus increased significantly when the samples were soaked in PBS. According to Duan et al. [29], the presence of water and ions in the PCL scaffold significantly impact the material’s ability to absorb mechanical energy. As a result, the buffer content can be viewed as an external variable that can influence the polymeric structure’s molecular mobility.

Finally, the absence of a linear dependence of mechanical characteristics on the amount of injected Col can be attributed to several factors. Firstly, the concentrations of Col used in the study may have been too low to induce significant changes in the fiber structure and the polymeric solution. Furthermore, it is important to consider that the PCL-Col C membranes exhibited a higher fiber diameter compared to other compositions. This variation in fiber diameter suggests that different concentrations of Col can result in varying fiber sizes, which can, in turn, impact the mechanical properties of the membranes. Thus, the lack of a linear relationship between the amount of Col and mechanical characteristics could be influenced by the diverse fiber diameters observed.

### 3.3. Thermogravimetric Analysis

**Figure 4:**
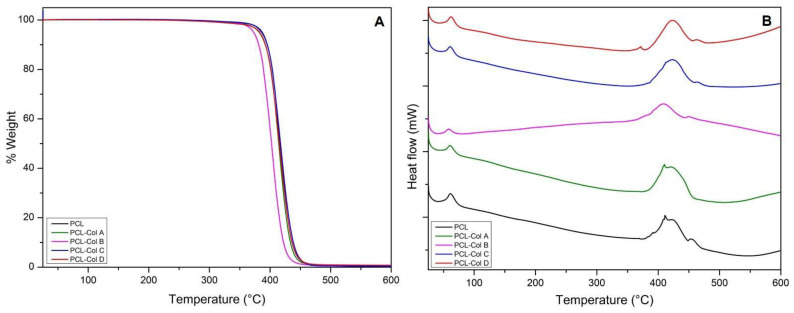
Characterization of PCL membranes loaded with Col at different concentrations; (**A**) thermogravimetric analysis and (**B**) differential scanning calorimetry.

Thermal analyses using DSC were conducted to examine the thermal properties of PCL scaffolds loaded with various concentrations of Col. The results obtained from the DSC and TGA curves are summarized in Table 2, and the corresponding diagrams can be seen in Figure 4. In all cases, the scaffolds exhibited a two-step degradation process.

The first endothermic peak’s maximum value was utilized to determine the melting temperature (T_m_), while the area under the curve of the Tm peak represented the melting enthalpy (ΔH_m_). Incorporating Col at different concentrations in the PCL scaffolds resulted in a reduction in the T_m_ of the scaffolds, decreasing from 61.40 °C to 34.65 °C. It is worth noting that PCL is a semicrystalline polymer with a typical T_m_ range of 59–60 °C [30]. Consistent with the findings reported by Wsoo et al. [31] following their incorporation of Col into other polymers like cellulose acetate, our study also observed a decrease in the polymer melting temperature (T_m_) when Col was incorporated into the PCL scaffolds.

Furthermore, an increase in the ΔH_m_ was observed upon the addition of Col to the PCL membranes. This could be attributed to an increase in hydrogen bond formation between the polymer and Col [32].

The crystallinity of the polymer was determined from the DSC analysis using Equation (3), where the reference value for 100% crystalline PCL was 139.3 J/g, and ΔH_m_ is the melting enthalpy value obtained from the first peak in the DSC.

(3) $$C_{x}=\frac{∆H_{m}}{139.3\;}$$

According to this calculation, it was found that the incorporation of Col resulted in an increase in the crystallinity of the polymer. This finding is consistent with the observations reported by Wsoo et al. [31], where an increase in crystallinity was attributed to the loss of nanofiber entanglement and potential recrystallization through thermal sintering near the melting point. Among the scaffolds, the PCL-Col A scaffold exhibited the highest crystallinity, while the PCL-Col B scaffold had the lowest.

A higher degree of crystallinity is typically associated with an ordered arrangement, which can enhance the material’s ability to withstand greater loads [33]. However, in this case, the presence of different Col concentrations in the PCL scaffolds resulted in varying fiber diameter distributions, which may affect their mechanical properties.

These observations suggest that, while increased crystallinity can confer improved load-bearing capabilities, the specific Col concentrations used in the PCL scaffolds may introduce variations in fiber diameter distribution, which could potentially influence their mechanical performance. 

In terms of degradation temperature (T_b_), it was observed that the samples PCL-Col C and PCL-Col D exhibited an increase from 410 °C to 422 °C. This increase suggests that PCL-Col D possesses greater thermal stability compared to PCL-Col C.

The higher T_b_ of PCL-Col D implies that the presence of Col at the specific concentration used in this sample enhances the thermal stability of the PCL scaffold. This finding suggests that the incorporation of Col can have a positive influence on the thermal properties and stability of the PCL-based scaffold.

The improved thermal stability of PCL-Col D may have practical implications in applications where thermal resistance is important, such as in the fabrication process or under conditions involving elevated temperatures. It indicates that PCL-Col D may be better suited for such scenarios, offering enhanced resistance to thermal degradation.

**Figure 5:**
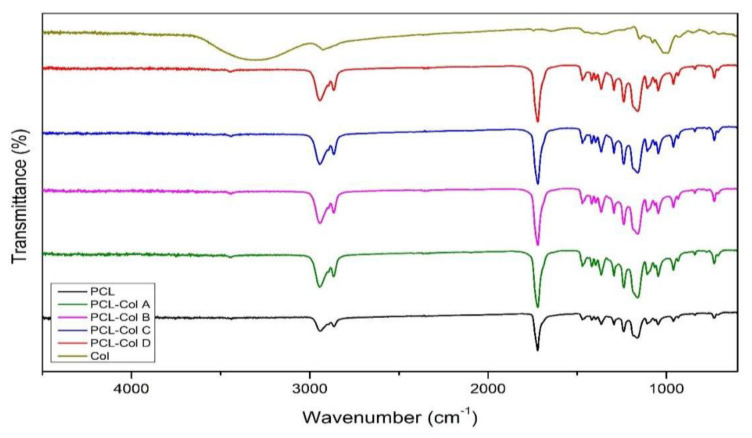
FT–IR of the different PCL scaffolds loaded with Col. In addition, PCL alone and free Col spectra are incorporated.

Figure 5 shows the FT-IR spectra of the PCL scaffolds loaded with the different concentrations of Col as well as the spectra of Col and PCL alone. All the scaffolds show a spectrum that is characteristic of PCL. An intense characteristic band of the PCL backbone was observed at 1720 cm^−1^, corresponding to a stretching ester group [34]. Bands at 2931 and 2852 cm^−1^ correspond to asymmetric and symmetric stretching -CH_2_, respectively [35]. No differences were noted in the FT-IR spectra of the scaffolds, regardless of the Col concentration. This may be due to the low concentration of the bioactive compound in the scaffolds. 

**Figure 6:**
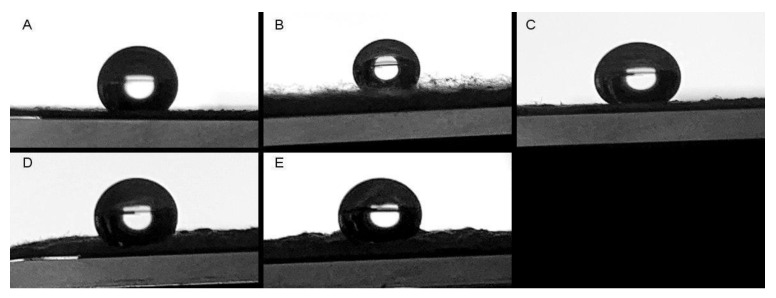
Static contact angle on the (**A**) PCL scaffolds, (**B**) PCL-Col A, (**C**) PCL-Col B, (**D**) PCL-Col C, and (**E**) PCL-Col D.

Furthermore, the water contact angle was measured on each membrane to assess their hydrophobic properties. All measured values exceeded 90°, indicating that all membranes exhibited superhydrophobic characteristics (Figure 6). Interestingly, no significant changes in the water contact angle were observed with varying Col concentrations, implying that the presence of Col did not impact the superhydrophobic nature of the PCL membranes. These findings align with previous reports by different authors who also reported similar contact angle values for PCL membranes [36,37,38].

### 3.4. Cell Biocompatibility Evaluation 

#### 3.4.1. Cell Proliferation

**Figure 7:**
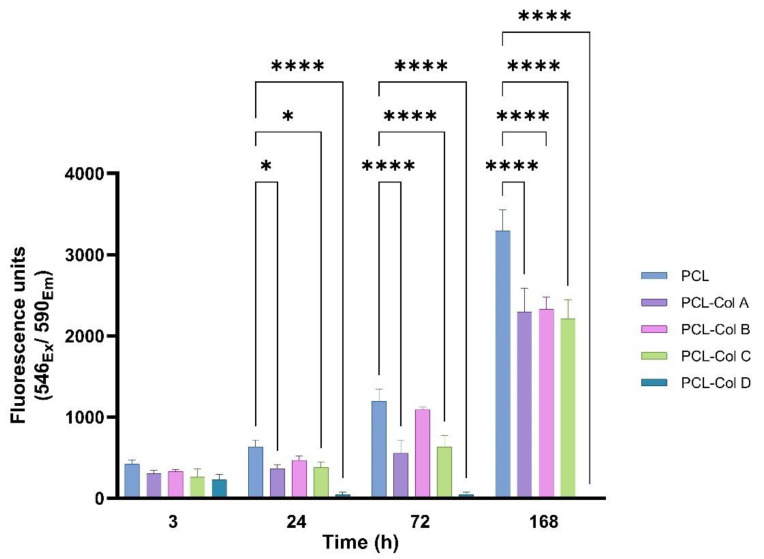
Fluorescence readings of the metabolic activity of MG-63 cultured on PCL scaffolds loaded with Col for 1, 3, and 7 days (* *p* < 0.05; **** represents a *p* < 0.0001).

The metabolic activity of MG-63 cells was measured using the Alamar Blue test after they were cultured on PCL scaffolds loaded with different Col concentrations for 1, 3, and 7 days (Figure 7). 

Cell viability was measured after 3 h of incubation to assess cell adhesion on the scaffolds. After 3 h of culture, there were no significant differences in cell adhesion, which is consistent with the results obtained from the water contact angle.

After 24 h, MG-63 cells proliferation was higher on the PCL without Col than on PCL-Col A, PCL-Col C, and PCL-Col D, which may suggest that the electrospun PCL scaffold did not affect the cell viability of fibroblasts at the evaluated time points, likely due to high surface-area-to-volume ratio of the membranes.

The initially attached cells died in PCL-Col D, and there was no cell proliferation over time. Furthermore, no stained cells were found via confocal microscopy.

These results contradict the findings reported by Sattary et al. [35], who observed higher cell viability in scaffolds containing Col compared to their other tested scaffolds. However, it is important to note that their study examined only one concentration of Col, and they reported a synergistic effect between the scaffolding material and Col.

#### 3.4.2. Cell Cytotoxicity

**Figure 8:**
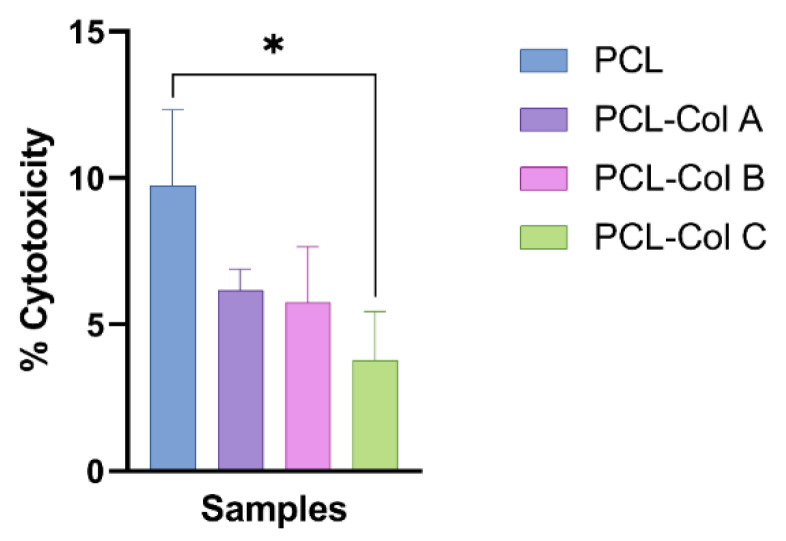
Percentage of LDH released to the medium by MG-63 cells after 24 h of incubation in contact with the scaffolds (* *p* < 0.05).

Lactate dehydrogenase is a cytosolic enzyme present in different cell types and a reliable indicator of cytotoxicity. The presence of LDH in the extracellular medium is generally correlated with cell membrane damage [39]. Thus, as the extracellular LDH activity measured in the culture medium was lower than the intracellular LDH activity measured after cell lysis, it was confirmed that the PCL scaffolds loaded with Col do not damage the scaffold (Figure 8).

**Figure 9:**
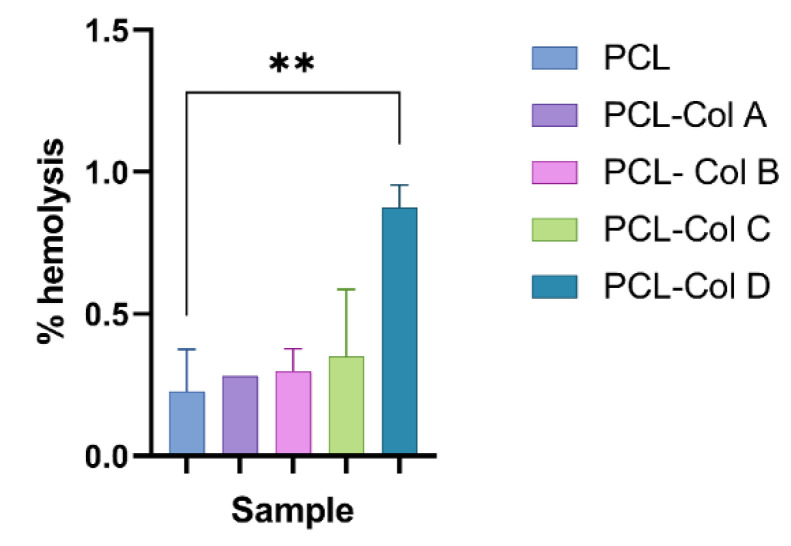
Hemolysis percentage of PCL membranes loaded with different concentrations of Col (** *p* < 0.01).

Hemolysis evaluation can be used as an index of hemocompatibility for materials intended for biological applications [17]. All the PCL membranes loaded with the different Col concentrations produced hemolysis values below 2% (Figure 9). According to ISO 10993-4, materials showing hemolysis values lower than 5% can be used as blood-contacting materials. Thus, these results indicated that, despite the PCL-Col D scaffolds producing a significantly higher percentage of hemolysis than the PCL scaffolds without bioactive, all the scaffolds evaluated in this study could be used as a blood-contacting material for tissue regeneration. 

#### 3.4.3. Bone Matrix Evaluation

**Figure 10:**
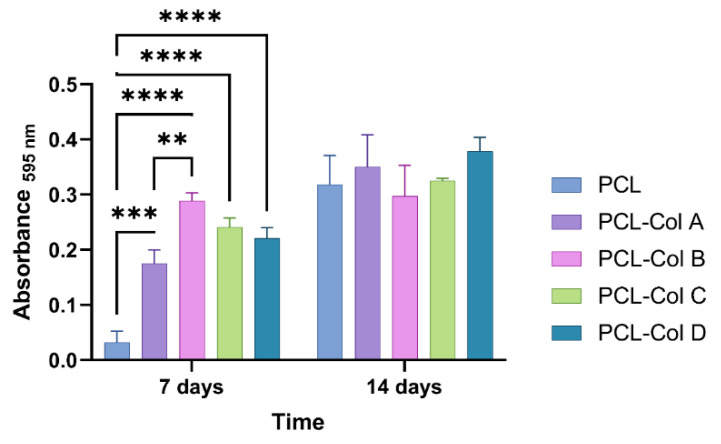
Quantitative analysis of alkaline phosphatase staining in the membranes after 7 and 14 days of incubation with MG-63 cells (** represents a *p* value < 0.01; *** represents a *p* value < 0.001, and **** represents a *p* < 0.0001).

Col is crucial for maintaining bone health and density, and it shows great potential in preventing and treating osteoporosis. It also plays an essential role in bone metabolism and is known to promote bone regeneration. Recent studies have explored the use of Col in electrospun membranes for bone regeneration. These studies have demonstrated that incorporating Col into the electrospun scaffolds can enhance the osteogenic differentiation of mesenchymal stem cells (MSCs) and increase bone formation in vivo [40,41]. In our study, the capability of the scaffolds to stimulate bone matrix formation was evaluated by investigating the expression of alkaline phosphatase (ALP) as an earlier indicator of matrix mineralization. Additionally, calcium fixation was measured by staining the membranes with Alizarin Red S (Figure 10). 

Briefly, MG-63 cells were incubated for 14 days on PCL scaffolds loaded with different concentrations of Col. Subsequently, 1 cm^2^ pieces of each scaffold were incubated under the same conditions without cells as a stain control. ALP activity was measured on days 7 and 14 to evaluate the expression of the enzyme over time. The results of this study suggests that the presence of Col in PCL scaffolds can significantly increase alkaline phosphatase expression in MG-63 cells after 7 days of incubation compared to cells cultured on PCL membranes without Col, which is important for bone and other connective tissue formation [42]. Specifically, PCL-Col B produced the highest expression of alkaline phosphatase after 7 days of culture. 

**Figure 11:**
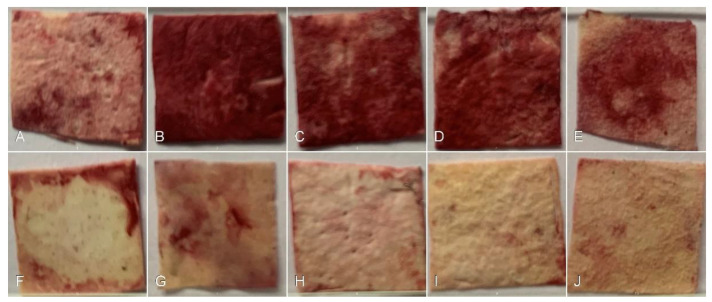
Images of PCL scaffold loaded with different concentrations of Col after Alizarin Red S staining and 7 days of culture with MG-63 cells. (**A**) PCL membrane without Col, (**B**) PCL-Col A, (**C**) PCL-Col B, (**D**) PCL-Col C, and (**E**) PCL-Col D scaffolds. Images (**F**–**J**) correspond to the control without cells of each sample.

The calcium-mineralized nodules on the scaffolds were stained using the Alizarin Red S assay. The results indicate that the presence of Col in the PCL-Col A scaffold leads to an increase in mineralization compared to the other samples tested. However, as the concentration of Col increases, there is a decrease in mineralization, which could be attributed to the lower viability of MG-63 cells (Figure 11).

In a recent study, Al-Bishari et al. [40] reported the production of PCL fibers incorporating curcumin and Col to enhance bone formation. They found that the incorporation of curcumin and Col resulted in the improved proliferation and differentiation of osteoblasts, along with increased alkaline phosphatase (ALP) activity. Similarly, Sattary et al. [43] developed a matrix consisting of PCL, gelatin, hydroxyapatite, and cholecalciferol. Their evaluation concluded that the addition of cholecalciferol had a more significant influence on ALP activity compared to the addition of nanohydroxyapatite, which can be attributed to increased calcium mineralization.

The increase in mineralization observed following the addition of Col to the PCL scaffold is a promising finding, suggesting that Col may enhance bone tissue formation. In line with this, Mason et al. [44] demonstrated that the use of Col resulted in increased alkaline phosphatase activity, indicating extracellular matrix (ECM) maturation and calcium deposition. However, the decrease in mineralization at higher concentrations of Col and the formation of hypercalcification spots in PCL-Col D suggest that there may be a threshold beyond which the presence of Col could negatively affect bone tissue formation. Further investigations are necessary to determine the optimal conditions for bone tissue engineering by examining the effect of different concentrations of Col on mineralization in vivo.

Finally, these findings may be useful in the design of bone implant materials or tissue engineering, as they suggest that including Col in PCL scaffolds can enhance the cells’ ability to produce alkaline phosphatase in the early stages of cell culture and induce calcium fixation into the membrane. However, further studies are needed to determine whether Col has any long-term effects on alkaline phosphatase expression or bone and connective tissue formation.

## 4. Conclusions

In conclusion, the resulting scaffolds had an average fiber diameter of 2–9 μm and high elongation and elasticity under dry and wet conditions. The scaffolds were hemocompatible and non-cytotoxic in the biocompatibility tests conducted on MG-63 cells. In addition, the present study demonstrates that incorporating Col into electrospun PCL scaffolds can enhance the expression of alkaline phosphatase in MG-63 cells and promote their mineralization. However, it was observed that higher concentrations of Col could decrease cell viability and produce hypercalcification spots.

These findings suggest that PCL-Col scaffolds have potential as biocompatible matrixes for bone tissue engineering in different fields of bone regeneration. However, further studies are required to investigate the efficacy of PCL-Col scaffolds in vivo, particularly in terms of bone regeneration and bone quality. Future research could also focus on optimizing the concentration of Col in the PCL scaffolds to enhance their osteogenic properties for use in vivo. Overall, the electrospinning technique used in this study provides a promising method for producing PCL-Col scaffolds with tailored properties for bone tissue engineering and could have important implications for developing new therapies for bone defects and injuries.

One promising direction for future research could be to investigate the potential of combining Col with other polymers to optimize bone tissue regeneration. Moreover, in vivo studies could be conducted to evaluate the efficacy of PCL scaffolds loaded with Col in promoting bone formation and integration with the surrounding tissue. Such studies could help determine the optimal conditions for incorporating Col into electrospun PCL scaffolds to ensure effective bone regeneration. Overall, this study provides valuable insights into the use of Col in electrospun PCL membranes for bone tissue engineering, highlighting the need for further research to fully understand its potential in clinical applications.

## 5. Patents

These results are protected by a provisional patent application to the World Intellectual Property Organization (WIPO); reference number 2020-63/477,297.

## Figures and Tables

**Table 1:** Mechanical characterization of dry and wet PCL scaffolds loaded with different Col concentrations.

		Elongation at Break (%)	Tensile Strength (Mpa)	Young’s Modulus
PCL	Dry	665.26 ± 49.10	3.10 ± 0.13	8.92 ± 0.30
	Wet	524.03 ± 134.26	25.21 ± 3.50	35.81 ± 18.26
PCL-Col A	Dry	616.70 ± 139.15	1.35 ± 0.36	0.97 ± 0.13
	Wet	364.62 ± 67.58	12.69 ± 0.83	12.24 ± 0.83
PCL-Col B	Dry	514.35 ± 124.76	1.11 ± 0.10	1.12 ± 0.07
	Wet	420.72 ± 29.78	12.79 ± 0.73	13.35 ± 0.97
PCL-Col C	Dry	1053.30 ± 186.28 *	2.75 ± 0.13	5.04 ± 0.81
	Wet	1024.36 ± 509.51 *	25.47 ± 2.61	24.99 ± 0.99
PCL-Col D	Dry	589.38 ± 56.48	1.56 ± 0.29	2.21 ± 0.93
	Wet	791.93 ± 328.53	15.75 ± 0.00	14.80 ± 3.31

(* represents a *p* value < 0.05).

**Table 2:** Thermal characterization and contact angle of PCL membranes loaded with different Col concentrations.

	ΔH_m_ (J/g)	T_m_ (°C)	C_x_ (%)	T_b_ (°C)	Contact Angle (°)
PCL	32.600	61.40	23.40	410.07	130.04 ± 3.32
PCL-Col A	109.705	34.65	78.78	410.05	124.22 ± 4.78
PCL-Col B	56.313	37.26	40.42	404.04	130.95 ± 6.26
PCL-Col C	87.702	37.98	62.95	422.95	132.22 ± 13.91
PCL-Col D	96.045	36.72	68.94	422.14	131.77 ± 10.13

ΔH_m_: melting enthalpy (J/g); T_m_: melting temperature (°C); C_x_: Crystallinity degree (%); T_b_: Boiling temperature (°C).

## Data Availability

Due to an ongoing patent process, the data is currently not accessible to the public.

## Supplementary Materials

The following supporting information can be downloaded at: https://www.mdpi.com/article/10.3390/pharmaceutics15071939/s1, Table S1: Electrospinning conditions were systematically tested to optimize fiber production for achieving a micrometric size. The following parameters were evaluated: The assessed parameters were PCL concentration in chloroform (8–18 %wt), voltage (15–20 kV), and distance between the needle and the collector (10–20 cm). The flow rate was constant at 2 mL/h.
