# Supplementary material for: Osteoinductive Electrospun Scaffold Based on PCL-Col as a Regenerative Therapy for Peri-Implantitis
Source: Pharmaceutics. 2023 Jul 12;15(7):1939. doi: 10.3390/pharmaceutics15071939 (PMC10386026; doi:10.3390/pharmaceutics15071939)
Supplement: Supplementary file 1 [file pharmaceutics-15-01939-s001.zip › pharmaceutics-2406049-supplementary.pdf]

**Supplementary Table S1**. Electrospinning conditions were systematically tested to optimize fiber production for achieving a micrometric size. The following parameters were evaluated: The assessed parameters were PCL concentration in chloroform (8 - 18 %wt) voltage (15 - 20 kV) and Distance between the needle and the collector (10 - 20 cm). The flow rate was constant at 2 mL/h.

| % PCL | Voltage + (kV) | Distance (cm) | Flow rate (mL/h) | Fiber diameter (µm) |
|---|---|---|---|---|
| 8 | 25 | 20 | 2 | NFF |
| 8 | 20 | 20 | 2 | NFF |
| 8 | 15 | 20 | 2 | NFF |
| 8 | 25 | 17 | 2 | NFF |
| 8 | 20 | 17 | 2 | NFF |
| 8 | 15 | 17 | 2 | NFF |
| 8 | 25 | 10 | 2 | NFF |
| 8 | 20 | 10 | 2 | NFF |
| 8 | 15 | 10 | 2 | NFF |
| 10 | 25 | 20 | 2 | NFF |
| 10 | 20 | 20 | 2 | NFF |
| 10 | 15 | 20 | 2 | NFF |
| 10 | 25 | 17 | 2 | NFF |
| 10 | 20 | 17 | 2 | NFF |
| 10 | 15 | 17 | 2 | NFF |
| 10 | 25 | 10 | 2 | NFF |
| 10 | 20 | 10 | 2 | NFF |
| 10 | 15 | 10 | 2 | NFF |
| 12 | 25 | 20 | 2 | 3.70 ± 1.43 |
| 12 | 20 | 20 | 2 | 2.81 ± 1.84 |
| 12 | 15 | 20 | 2 | 4.94 ± 0.95 |
| 12 | 25 | 15 | 2 | NFF |
| 12 | 20 | 15 | 2 | NFF |
| 12 | 15 | 15 | 2 | NFF |
| 12 | 25 | 10 | 2 | NFF |
| 12 | 20 | 10 | 2 | NFF |

| | | | | |
|---|---|---|---|---|
| 12 | 15 | 10 | 2 | NFF |
| 14 | 25 | 20 | 2 | 4.76 ± 1.86 |
| 14 | 20 | 20 | 2 | 4.99 ± 1.95 |
| 14 | 15 | 20 | 2 | 5.39 ± 2.01 |
| 14 | 25 | 15 | 2 | 4.68 ± 1.21 |
| 14 | 20 | 15 | 2 | 2.68 ± 1.24 |
| 14 | 15 | 15 | 2 | 4.20 ± 0.97 |
| 14 | 25 | 10 | 2 | 3.78 ± 0.43 |
| 14 | 20 | 10 | 2 | 4.22 ± 0.59 |
| 14 | 15 | 10 | 2 | 4.28 ± 0.82 |
| 16 | 25 | 20 | 2 | 6.54 ± 1.13 |
| 16 | 20 | 20 | 2 | 4.99 ± 4.36 |
| 16 | 15 | 20 | 2 | 9.05 ± 2.39 |
| 16 | 25 | 17 | 2 | 4.64 ± 0.35 |
| 16 | 20 | 17 | 2 | 1.74 ± 0.44 |
| 16 | 15 | 17 | 2 | 0.76 ± 0.40 |
| 16 | 25 | 10 | 2 | 3.42 ± 1.56 |
| 16 | 20 | 10 | 2 | 5.01 ± 0.32 |
| 16 | 15 | 10 | 2 | 6.04 ± 0.46 |
| 18 | 25 | 20 | 2 | 7.11 ± 0.42 |
| 18 | 20 | 20 | 2 | 6.95 ± 0.65 |
| 18 | 15 | 20 | 2 | 6.72 ± 1.27 |
| 18 | 25 | 17 | 2 | 6.33 ± 0.56 |
| 18 | 20 | 17 | 2 | 6.79 ± 0.89 |
| 18 | 15 | 17 | 2 | 6.79 ± 1.92 |
| 18 | 25 | 10 | 2 | 4.10 ± 0.28 |
| 18 | 20 | 10 | 2 | 5.31 ± 0.38 |
| 18 | 15 | 10 | 2 | 6.66 ± 0.44 |

NFF: No fibers formation.
